# Immunogenicity of human embryonic stem cell-derived beta cells

**DOI:** 10.1007/s00125-016-4125-y

**Published:** 2016-10-27

**Authors:** Cornelis R. van der Torren, Arnaud Zaldumbide, Gaby Duinkerken, Simone H. Brand-Schaaf, Mark Peakman, Geert Stangé, Laura Martinson, Evert Kroon, Eugene P. Brandon, Daniel Pipeleers, Bart O. Roep

**Affiliations:** 1 Department of Immunohaematology and Blood Transfusion, E3-Q, Leiden University Medical Center, P.O. Box 9600, NL-2300 RC Leiden, the Netherlands; 2 JDRF Center for Beta Cell Therapy in Diabetes,; 3 Department of Molecular Cell Biology, Leiden University Medical Center, Leiden, the Netherlands; 4 Department of Immunobiology, King’s College London School of Medicine, London, UK; 5 Diabetes Research Center, Brussels Free University-VUB, Brussels, Belgium; 6 ViaCyte, Inc., San Diego, CA USA; 7 Department of Diabetes Immunology, Diabetes and Metabolism Research Institute at the Beckman Research Institute, City of Hope, Duarte, CA USA

**Keywords:** Allograft rejection, Autoimmunity, Beta cells, Embryonic stem cells, Transplantation

## Abstract

**Aims/hypothesis:**

To overcome the donor shortage in the treatment of advanced type 1 diabetes by islet transplantation, human embryonic stem cells (hESCs) show great potential as an unlimited alternative source of beta cells. hESCs may have immune privileged properties and it is important to determine whether these properties are preserved in hESC-derived cells.

**Methods:**

We comprehensively investigated interactions of both innate and adaptive auto- and allo-immunity with hESC-derived pancreatic progenitor cells and hESC-derived endocrine cells, retrieved after in-vivo differentiation in capsules in the subcutis of mice.

**Results:**

We found that hESC-derived pancreatic endodermal cells expressed relatively low levels of HLA endorsing protection from specific immune responses. HLA was upregulated when exposed to IFNγ, making these endocrine progenitor cells vulnerable to cytotoxic T cells and alloreactive antibodies. In vivo-differentiated endocrine cells were protected from complement, but expressed more HLA and were targets for alloreactive antibody-dependent cellular cytotoxicity and alloreactive cytotoxic T cells. After HLA compatibility was provided by transduction with HLA-A2, preproinsulin-specific T cells killed insulin-producing cells.

**Conclusions/interpretation:**

hESC-derived pancreatic progenitors are hypoimmunogenic, while in vivo-differentiated endocrine cells represent mature targets for adaptive immune responses. Our data support the need for immune intervention in transplantation of hESC-derived pancreatic progenitors. Cell-impermeable macro-encapsulation may suffice.

**Electronic supplementary material:**

The online version of this article (doi:10.1007/s00125-016-4125-y) contains peer-reviewed but unedited supplementary material, which is available to authorised users.

## Introduction

Beta cell replacement by islet transplantation can functionally cure long-standing type 1 diabetes but its implementation is limited by the lack of donor organs, loss of graft function over time and the side effects of obligatory immunosuppression. Alternative sources of beta cells could overcome the shortage of human islets, while novel protective strategies and/or the potential for immune privilege of an alternative source of beta cells may help to overcome the immunosuppressive burden.

Several alternative sources of beta cells are currently being explored, including human embryonic stem cells (hESCs) [1], proliferating beta cell lines [2], induced pluripotent stem cells [3, 4] and xenogeneic islets [5, 6]. hESC-derived beta cells are well on the way to clinical translation with recent publications on improved safety and scaling of a protocol being made by Kroon and colleagues [1, 7, 8].

After transplantation, hESC-derived beta cells face the challenges posed by the human immune system for the first time. Partial early loss of grafts through innate immune reactions may be overcome by transplanting more cells or by choosing an alternative site to the liver. However, both recurrent autoimmunity and alloreactive responses remain a persistent threat to transplanted human-derived beta cells despite immunosuppression [9–11]. Additionally, alloreactive responses provoked by a graft can be a risk for future transplantations [12–14]. Conversely, complete lack of immune interaction can leave cells vulnerable to infections or may invoke an innate immune attack by natural killer (NK) cells [15]. We recently showed this for immortalised beta cell lines [16].

Embryonic stem cells have immune privileged properties and can resist alloreactive responses [17]. However, differentiation of hESCs to other cell types can result in the loss of this immunological privilege [17–21]. Insights into the immunogenicity of the alternative sources of beta cells can help to guide the choice of immune-protective strategies and the relevant immune monitoring to be employed in clinical introduction. We therefore investigated the immunogenicity of hESC-derived beta cells and their progenitors to adaptive immune responses relevant in transplantation.

## Methods

### Biological reagents

hESC-derived pancreatic endodermal cells (hESC-PEs) were prepared from the CyT49 cell line (HLA A1, A3, B44, B57) at ViaCyte (San Diego, CA, USA), cryopreserved and shipped to Brussels, as described [22]. For each series of experiments, cells were thawed and cultured for 72 h in db-N50-K50-E50 medium [7]. A fraction of the cells was sent to Leiden in db medium [1] and the remaining cells were transplanted in Brussels. Encapsulated grafts were prepared by loading 4 × 10^6^ cells in a macro-device (ViaCyte) and were implanted in the subcutis of non-diabetic NOD/severe combined immunodeficiency (SCID) male mice (7–8 weeks old, NOD.CB17-*Prkdc*scid/J; Charles River, L’Arbresle, France). Implants were analysed in vivo and ex vivo as described [22]. In vivo graft function was followed over 20 weeks through measuring plasma human C-peptide levels 15 min after intraperitoneal injection of glucose; at post-transplant week 20, all recipients were confirmed to have reached plasma human C-peptide levels above 1 ng/ml. Implants were resected at post-transplant week 20–25 and dispersed for analysis before sending to Leiden in phosphate-buffered Ham’s F-10 medium (Gibco, Bleiswijk, the Netherlands), supplemented with 0.5% human albumin, 2 mmol/l l-leucine and 2 mmol/l l-glutamine. Upon arrival, hESC-PEs and in vivo-differentiated cells were dissociated with 0.05% trypsin-EDTA 1X (Gibco) to single cells for immunological assays.

Human peripheral blood mononuclear cells (PBMCs) were separated from whole blood (for T cells) or buffy coats (for lymphocytes) by Ficoll–Hypaque density gradient. Peripheral blood lymphocytes were left over after CD14 depletion from PBMCs with CD14-MicroBeads according to the manufacturer’s protocol (Miltenyi Biotec, Auburn, CA, USA). Preproinsulin (PPI)-specific T cell clone 1E6 generation was described previously [23]. Briefly, PBMCs from an individual with type 1 diabetes were stimulated with PPI_15-24_ peptide. PPI-specific cytotoxic T lymphocytes (CTLs) were sorted by FACS and expanded. Alloreactive CTL clones JS132 (to HLA-A2) and C776 (to HLA-A1) were generated by stimulating PBMCs, collected from a healthy donor, with irradiated Epstein–Barr virus (EBV)-transformed B cell line expressing the HLA of interest. After several rounds of stimulation and enrichment, the alloreactive population was cloned by limiting dilution at 0.5 cell/well [24]. CTL clones to cytomegalovirus (CMV) peptides VTEHDTLLY in HLA-A1 (clone 3c8) and NLVPMVATV in HLA-A2 (clone 18) were generated by single cell sorting of CD8^+^ T cells stained with the respective tetramers and expanded using phytohaemagglutinin stimulation [25]. HEK-293 and primary tubular epithelial cells (PTECs, line HK-2) were cultured in DMEM/F-12 medium (Invitrogen, Landsmeer, the Netherlands) supplemented with 2 mmol/l l-glutamine, 25 mmol/l HEPES, 50 U/ml penicillin and 50 μg/ml streptomycin (all purchased from Invitrogen) and, for PTECs, also 5 μg/ml insulin, 5 μg/ml transferrin, 5 ng/ml selenium, 36 ng/ml hydrocortisone and 10 ng/ml epidermal growth factor (all purchased from Sigma, Zwijndrecht, the Netherlands). B lymphocytes (B-LCL) lines were cultured in IMDM (Invitrogen) supplemented with 2 mmol/l l-glutamine, 25 mmol/l HEPES, 50 U/ml penicillin, 50 μg/ml streptomycin and 5% FBS.

Human monoclonal antibodies recognising HLA-A68 or HLA-B8 were selected from a panel as described previously [26]. In short, heterohybridomas were created by EBV transformation and cloning of B-LCL of multiparous women. The HLA specificities of the produced human monoclonal antibodies were validated using a complement-dependent cytotoxicity test on PBMCs.

Beta cell-specific T helper (Th) cell supernatant fractions were generated by incubating islet preparation reactive Th1 clone (1c6) from a diabetic patient with HLA-matched PBMCs pre-incubated with or without 10 μg/ml antigen in RPMI 1640 medium (Gibco) mixed without and with 11 mmol/l glucose to achieve 5.6 mmol/l d-glucose and supplemented with 2 mmol/l glutamine (Gibco) [27]. After 3 days the supernatant fraction was harvested and frozen until use.

For compatibility with autoreactive CTL clone 1E6, beta cell line EndoC-βH1 was transduced with a lentiviral vector containing HLA-A02:01 under elongation factor 1α (EF1α) promotor at multiplicity of infection of 2. Third-generation self-inactivating lentivirus vectors were produced as described previously [28]. The generation of human cell lines and antibodies was carried out after obtaining informed consent and with approval of the institutional review board, in accordance with the 2008 revised principles of the Declaration of Helsinki.

### Alloreactivity assays

Alloreactive cellular cytotoxicity was assessed by chromium release (PerkinElmer, Waltham, MA, USA). Briefly, dispersed hESC-derived cells were labelled with ^51^Cr for 60 min, washed three times and incubated with alloreactive T cells or, for antibody-dependent cellular cytotoxicity (ADCC), human monoclonal antibodies and peripheral blood lymphocytes in different effector-to-target ratios for 4–6 h or overnight. ^51^Cr release in supernatant fractions was assessed on a WIZARD2 γ-counter (Perkin Elmer, Waltham, MA, USA). Specific lysis was calculated as [(experimental release − spontaneous release) / (max release − spontaneous release)] × 100%.

Complement-dependent cytotoxicity was assessed with hESC-derived cells as targets in a clinical cross match assay [29]. In short, dispersed cells were incubated with serum containing alloreactive antibodies with known specificity and incubated in triplicate in Therasaki plates at room temperature for 1 h. Rabbit complement (Inno-train, Frankfurt am Main, Germany) was added and the plates were incubated for another hour. Cell lysis was assessed by adding propidium iodide–ink solution and measured on the Patimed system (Leica, Rijswijk, the Netherlands). Minimal and maximum lysis was set to HLA-antibody-negative serum cell death and parallel lysis of HLA-matched lymphocyte targets, respectively.

### Autoreactive assays

At 20 h post transduction, virus supernatant fraction was removed from HLA-A02:01 transduced cells by centrifugation. The cells were cultured in Ham’s F-10 medium (Gibco) supplemented with 200 mmol/l glutamine (Gibco), 0.5% BSA fraction V (Sigma-Aldrich, Zwijndrecht, the Netherlands), 1 mol/l CaCl_2_ and 1.8 g/l d-glucose for 4 days to allow transgene expression before trypsin dispersion and incubation with PPI-specific CTL clone 1E6 or CMV-specific clone 18 in a 1:5 ratio. After overnight incubation, cells were stained with Fixable Viability Dye eFluor 450 (eBioscience, Vienna, Austria), CD45-PerCP (2D1; BD, Breda, the Netherlands) and HLA-A2–fluorescein isothiocyanate (FITC) (BD), then fixed in paraformaldehyde and permeabilised with saponin before staining with guinea pig anti-insulin (Free University Brussels, Brussels, Belgium) and donkey anti-guinea pig Alexa 647 (Jackson Immunoresearch Laboratories, West Grove, PA, USA). Cells were measured on a CANTO II flow cytometer (BD). Transduction efficiency was determined by staining with anti-HLA-A2–Allophycocyanin (APC) (Bb7.2; BD) on a Calibur flow cytometer (BD).

### Cell surface staining

Cell surface antigens were analysed by FACS on a BD FACSCalibur after 20 min staining at 4°C with antibodies to HLA class I–FITC (W6/32; BD), HLA-DR–FITC (L243; BD), IgG1–APC (MOPC-21; BD), IgG2a–FITC (G155-178; BD), IgG2a–Phycoerythrin (G155-178; BD), anti-CD46–APC (MEM-258; ImmunoTools, Friesoythe, Germany), anti-CD55–Phycoerythrin (IA10; BD) and anti-CD59–APC (OV9A2, eBioscience).

## Experimental conditions

Data were excluded if positive or negative controls failed. Additional intermediate titrations were performed in parallel in most samples that were left out for clarity to the reader. These data supported the conclusion. Cell lines for comparison were available in our laboratory unless stated otherwise and mycoplasma contamination was regularly excluded. Non-commercial antibodies had been generated and validated in our department before [26] and were of IgG subclass.

### Statistical analysis

Data are represented as mean and SE unless stated otherwise. GraphPad Prism 6.0 (GraphPad Software, La Jolla, CA, USA) was used to create graphs and perform analysis. Student’s *t* test was used to compare continuous data and Fisher’s exact test was used for binominal data; *p* < 0.05 was considered statistically significant. All immune assays were replicated three times.

## Results

**Fig. 1:**
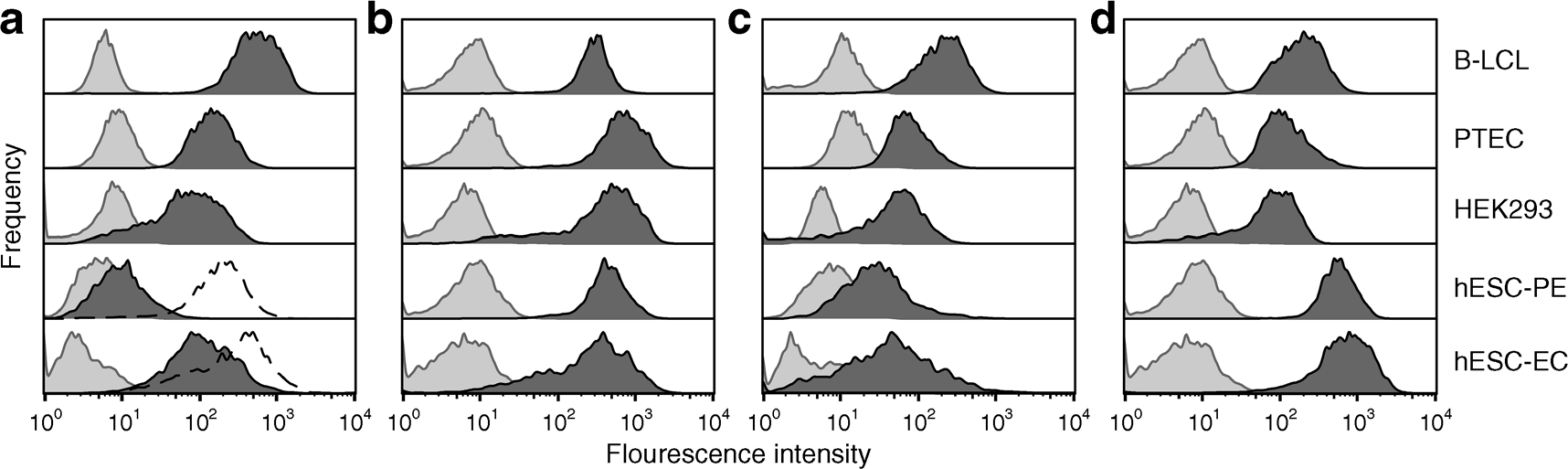
Expression of HLA and complement receptors. FACS analysis of HLA class I (**a**) and complement inhibitory receptors CD46 (MCP) (**b**), CD55 (DAF) (**c**) and CD59 (MIP) (**d**) expression on trypsin-dissociated hESC-PEs and differentiated hESC-ECs, compared with EBV-immortalised B-LCL, PTECs and HEK293 cells. HLA class I was upregulated by IFNγ (1000 IU/ml; dashed line). Light traces represent isotype control staining

Cell composition and function was assessed in hESC-PEs and cells retrieved from implants (hESC-derived endocrine cells [hESC-ECs]). These results and comparison with human islets have been reported [22]. Here, expression of HLA was assessed on the cell surface of hESC-PEs and hESC-ECs. This expression is essential for autoreactive, virus-specific T cell and alloreactive immune responses. hESC-PEs expressed very low levels of HLA class I, although HLA class I could be upregulated to reach levels expressed by other cell lines after exposure to the inflammatory cytokine, IFNγ (Fig. 1a). On the other hand, the hESC-ECs expressed normal levels of HLA class I, which were only slightly upregulated by IFNγ. HLA class II is generally not expressed on endocrine cells. In line with this only minimal expression over isotype control was noted for hESC-ECs on 3.5% of cells (and on 7.7% of cells after upregulation with IFNγ). Also, expression of complement receptors, which protect cells from complement-mediated destruction, was assessed. Both hESC-PEs and hESC-ECs expressed membrane cofactor protein (MCP; CD46) at the level of other cell lines (Fig. 1b), together with high levels of membrane attack complex-inhibitory protein (MIP; CD59) (Fig. 1d). The differentiated hESC-ECs clearly expressed decay-accelerating factor (DAF; CD55), similar to PTEC line HK-2 and HEK293 cells, while this expression was lower on hESC-PEs (Fig. 1c).

**Fig. 2:**
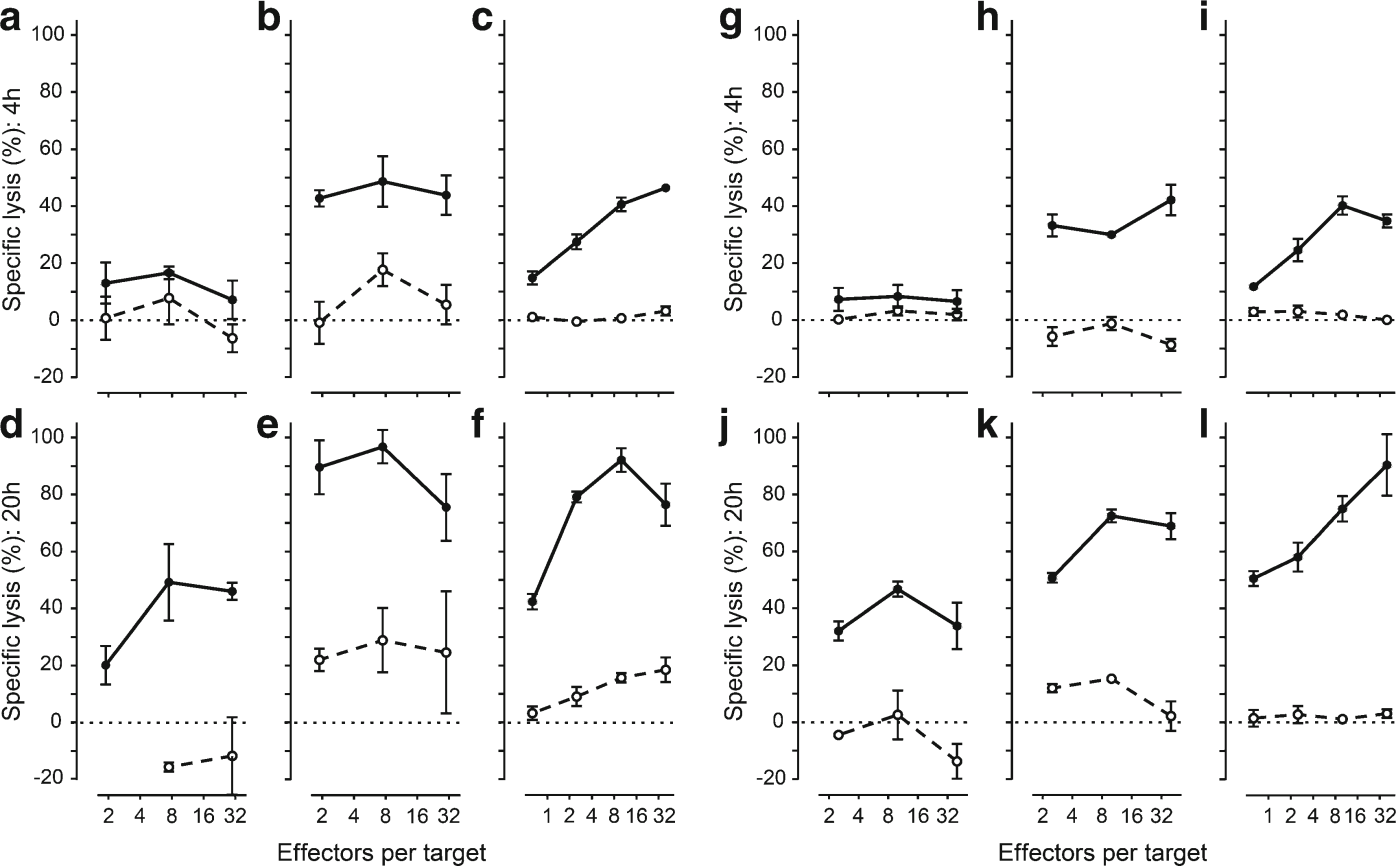
Alloreactive and virus-specific CTLs can target hESC-PEs and differentiated hESC-ECs. ESC-PEs (**a**, **b**, **d**, **e**, **g**, **h**, **j**, **k**) and hESC-ECs (**c**, **f**, **i**, **l**) expressing HLA-A1 were labelled with ^51^Cr and incubated with alloreactive CTLs targeting HLA-A1 (black circles, solid line) or targeting third party HLA-A2 (white circles, dashed line) (**a**–**f**) and virus-specific CTLs recognising CMV peptide in HLA-A1 on peptide-pulsed cells (black circles, solid line) or without peptide (white circles, dashed line) (**g**–**l**). Specific lysis after 4 h (**a**–**c**, **g**–**i**) and 20 h (**d**–**f**, **j**–**l**) was calculated relative to spontaneous lysis without T cells and chemically -induced maximum lysis. Inflammation was mimicked (**b**, **e**, **h**, **k**) by pre-incubation with IFNγ (1000 IU/ml), which upregulated HLA expression. Statistical results are available in ESM Fig. 1

Next, hESC-derived cells were tested for resistance or sensitivity to cytotoxic T cells. The hESC-PE was resistant to alloreactive CTLs directed against the specific HLA expressed by the nESC-PE in a 4 h chromium release assay (*p* > 0.05), but became vulnerable to the CTLs after exposure to IFNγ, which had upregulated HLA (*p* = 0.0005; Fig. 2).

**Fig. 3:**
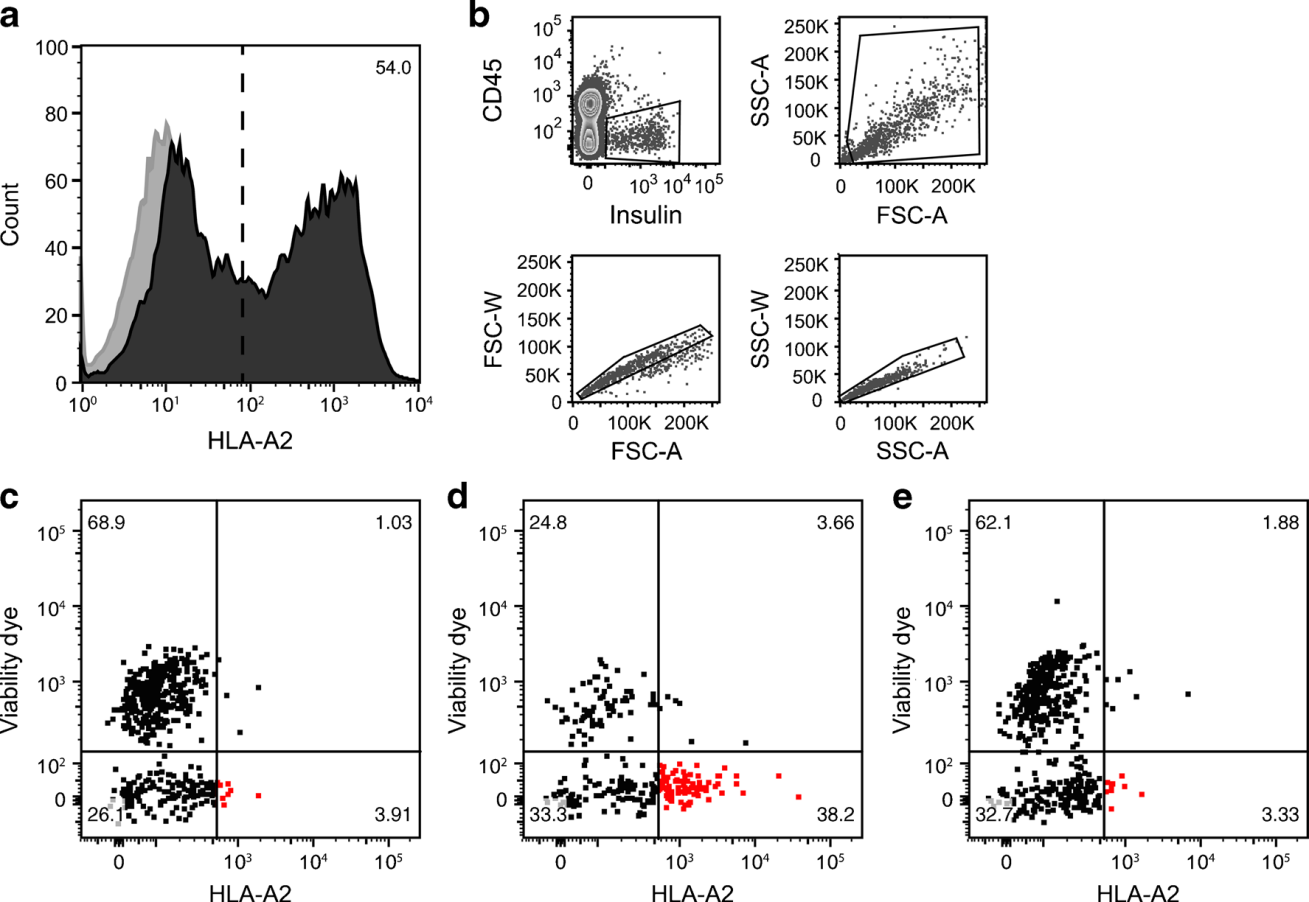
PPI-specific CTLs recognise and kill hESC-derived beta cells. (**a**) hESC-ECs were transduced with HLA-A2 for compatibility with autoreactive CTL clone 1E6. FACS analysis of HLA-A2 expression showed expression on >50% of hESC-ECs (dark grey; isotype control in light grey). (**b**) After 4 days’ upregulation of the transgene, hESC-derived cells were incubated with autoreactive CTL clone 1E6 recognising PPI peptide in HLA-A2 or clone 18 recognising an irrelevant CMV peptide for 20 h. Specific cytotoxicity was assessed by FACS by gating insulin-positive, CD45-negative, single cells. (**c**, **d**) Gated beta cell-like cells expressing HLA-A2 (red) were killed overnight with >90% efficiency by PPI-specific CTLs (**c**) compared with non-specific CTLs recognising CMV peptide (**d**). (**e**) The killing efficiency may be at maximum since this could not be increased by pulsing the beta cell-like cells with PPI peptide before exposure to PPI-specific CTLs. FSC-A, forward scatter: area; FSC-W, forward scatter: width; SSC-A, side scatter: area

To test recognition by memory autoreactive CTLs, HLA matching had to be introduced. Beta cell-specific autoreactive memory CTLs of type 1 diabetes patients pose a particular threat to transplanted beta cells if transplanted beta cells match recipient HLA. HLA-A2 was lentivirally introduced in hESC-ECs under the constitutive EF1α promoter (55% transduction efficiency). It was thus found that hESC-ECs expressing insulin and HLA-A2 were specifically killed by PPI-specific CTLs with > 90% efficiency when compared with non-specific CTLs (*p* < 0.0001; Fig. 3). The viability of mock-transduced insulin-expressing hESC-ECs incubated with PPI CTLs was comparable with that of transduced cells incubated with non-specific CTLs (73.2 and 71.5%, respectively). Adding PPI-positive control peptide did not further increase killing of insulin-expressing cells, but did lead to recognition and killing of HLA-A2 transduced cells that did not express insulin (data not shown).

To confirm peptide-specific killing of hESC-derived cells through their cognate HLA, we assessed killing by CTLs recognising CMV peptide in cognate HLA-A1. Similar to alloreactive CTLs, CMV-specific CTLs did not effectively kill hESC-PEs loaded with CMV peptide unless the hESC-PEs were exposed to IFNγ or the assay was prolonged. The differentiated hESC-ECs were vulnerable to alloreactive and CMV-specific CTLs without prior IFNγ treatment, while prolonged exposure increased killing to > 90% (Fig. 2).

**Fig. 4:**
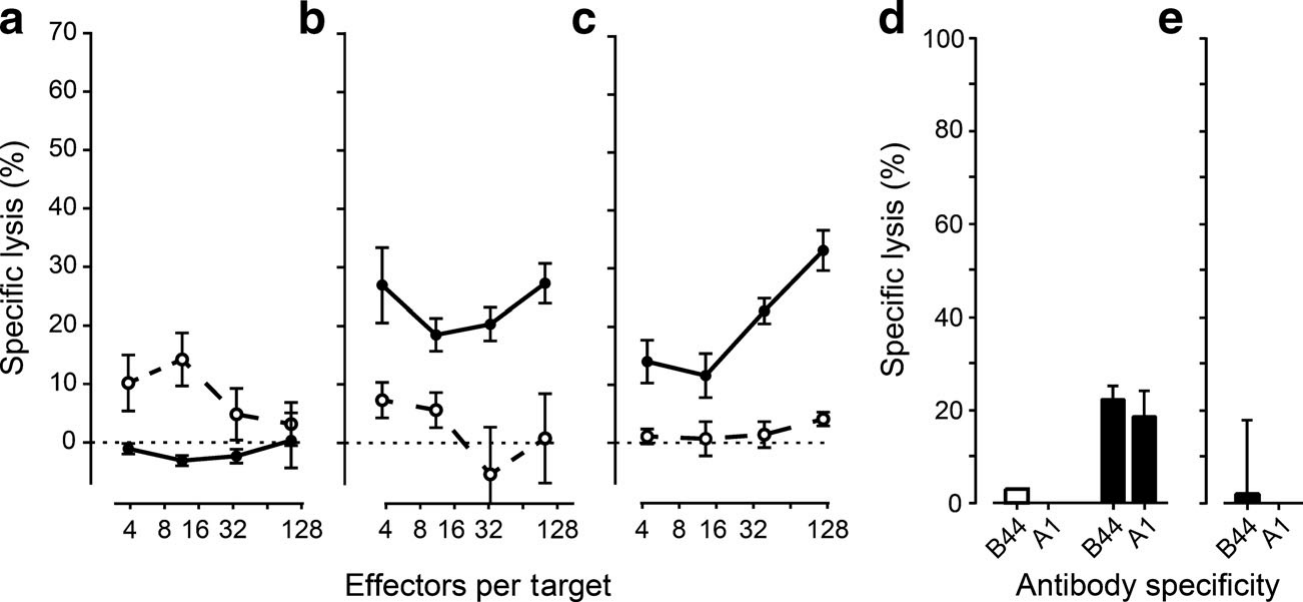
Alloreactive antibodies induce cellular cytotoxicity but differentiated hESC-ECs resist complement-dependent cytotoxicity. (**a**–**c**) hESC-PEs (**a**, **b**) and differentiated hESC-ECs (**c**) were labelled with ^51^Cr and incubated with specific (black circles, solid line) and non-specific (white circles, dashed line) alloreactive antibodies and peripheral blood lymphocytes for 6 h. Chromium release was measured and specific lysis was calculated relative to spontaneous lysis without T cells and chemical induced maximum lysis. Inflammation was mimicked by pre-incubation with IFNγ (1000 IU/ml), upregulating HLA expression (**b**). (**d**, **e**) hESC-PEs (**d**) and hESC-ECs (**e**) were incubated with serum containing HLA specific antibodies to A1 and B44 for 1 h and then for 1 h with rabbit complement. Lysis was assessed by propidium iodide staining with automated microscopy measurement and specific lysis calculated relative to cell death without specific antibodies and parallel specific lysis of lymphocyte controls. Inflammation was mimicked by pre-incubation with IFNγ (1000 IU/ml), upregulating HLA expression (black bars); white bars, no pre-incubation with IFNγ. hESC-ECs were only tested after IFNγ pre-incubation. Statistical results are available in ESM Fig. 2

Alloreactive antibodies can lead to rejection of transplants through the induction of ADCC or activation of the complement cascade. Human antibodies specific for the HLA of the hESC-derived cells or a third party were used to induce cytotoxicity by peripheral blood lymphocytes or by rabbit complement. hESC-PEs were not sensitive to alloreactive antibody-induced cytotoxicity unless HLA was upregulated by IFNγ (Fig. 4). In vivo-differentiated hESC-ECs exhibited an increased sensitivity to antibody-induced cellular cytotoxicity without prior inflammatory HLA upregulation, but not to complement-dependent cytotoxicity, regardless of IFNγ upregulation of HLA (Fig. 4).

## Discussion

Insights into the immunogenicity of beta cell-like cells from sources that are alternatives to human cadaver islets will help the decision-making process when choosing immune-protective strategies to test and the most relevant immune monitoring to perform in clinical translation. hESC-PEs are promising candidates for clinical application [30]. We therefore investigated the immunological properties of hESC-derived pancreatic cell preparations before and after differentiation to endocrine cells. We found that hESC-PEs maintain immune privilege with low HLA expression, although upregulation appears possible in inflammatory conditions which then render the cells vulnerable to adaptive immune responses. In vivo differentiation of hESC-PEs into a preparation with pancreatic endocrine cells also leads to immunological differentiation with increasing HLA expression and sensitivity to adaptive immune responses and also increased resistance to complement-mediated attack. Recognition and destruction by autoreactive PPI-specific T cells verified mature beta cell immune presentation by hESC-ECs.

Low expression of HLA by hESCs and hESC-derived cells underlies the hypoimmunogenicity observed in most published studies, effectively hiding cells from T cell recognition and alloreactive antibodies, while differentiation can upregulate HLA and increase vulnerability to immunity [17–21]. In our study, immunogenicity to alloreactive and peptide (CMV)-specific responses was related to HLA expression. hESC-PEs expressed very low levels of HLA in a non-inflamed environment, making them hypoimmunogenic, while differentiation to hESC-ECs resulted in an increase in HLA expression and immunogenicity to T cells to levels reported for human islets in the literature [23, 31–34].

Despite the hypoimmunogenicity of hESC-PEs, resistance was incomplete after prolonged exposure to high numbers of CTLs. This suggests that hESC-PEs do not employ active suppression of CTL responses, as has been observed in other studies [21]. Further, HLA expression could be induced by mimicking inflammation with IFNγ. While this increased immunogenicity to alloreactive T cells and antibodies, the incomplete immune privilege may help to ensure adequate virus control in case of infection and prevent destruction by inflammation-activated NK cells [35].

Increased expression of HLA through inflammation or differentiation of hESC to beta cells may make cells vulnerable to recurrent autoimmunity if cells are transplanted to an HLA-matched individual with type 1 diabetes. After realising HLA match through transduction of in vivo-differentiated hESC-ECs, the insulin-expressing cells could be selectively eliminated by autoreactive CTLs previously isolated from a donor with type 1 diabetes [23]. Thus, we show hESC-derived beta cells risk recurrent autoimmunity in an HLA-matched setting. Further, by proving presentation of the PPI peptide recognised by the CTL clone, we verify that hESC-ECs have active cellular mechanisms in place to ensure immune surveillance and that they display genuine insulin production allowing natural presentation of PPI epitopes in HLA.

In vivo differentiation of hESC-PEs to hESC-ECs increased resistance to complement-mediated attack despite increased expression of antigen (HLA) recognised by alloreactive antibodies. Protection from complement is mediated through complement inhibitory receptors, such as CD46 (MCP), CD55 (DAF) and CD59 (MIP), which are known to be expressed by human islets [36, 37]. All three receptors were expressed by the hESC-derived cells but CD55 expression was low on hESC-PEs, which may explain their vulnerability to complement-mediated cytotoxicity.

Although we were able to study diverse immune mechanisms relevant for transplantation of alternative beta cells, extrapolation of these results to the clinical transplantation setting is limited by several factors. First, animal models for in vivo differentiation of hESC-PEs to hESC-ECs may not completely reflect the differentiation process in humans. Second, culture conditions that are required for investigation of explanted in vivo-differentiated cells may alter the properties of the cells or the composition of the population. To this end, staining for beta cells did not show selective reduction of beta cells. Incomplete dissociation of naturally aggregating hESC-derived pancreatic endodermal cells and endocrine cells required for immune assays may contribute to the variation and relatively large standard error in the cytotoxicity assays (Figs 2, 4).

A head-to-head comparison with primary human islet cells would be of interest and was considered when setting up this study. However, it proved to be logistically impossible. hESCs require 4 months of in vivo differentiation into endocrine cells and the ex vivo experiments must be started within 24 h after explantation to assure optimal cell quality. The supply of fresh human islets for research purposes is highly infrequent and rare, precluding parallel testing with differentiated hESC-ECs. This was limited further by the HLA restrictions enforced by this type of experiment. We therefore looked to data on beta cell immunogenicity from the literature for the purpose of comparison [23, 31–34, 36].

The identified immunogenicity of hESC-derived pancreatic endocrine cells highlights the need for an immune suppressive strategy for human transplantation. The vulnerability of these hESC-derived cells to cellular attack and their resistance to complement-mediated attack suggests that providing protection by cell-impermeable macro-encapsulation could be a successful strategy in clinical implementation. Alternatively, or additionally, the initial hypoimmunogenicity of the engrafted pancreatic endoderm may provide a window of opportunity in which to induce graft-specific tolerance.

In conclusion, pancreatic endoderm progenitors maintain hypoimmunogenicity, while differentiation of hESCs to pancreatic endocrine cells increases vulnerability to cellular immunity. Inflammatory conditions further increase immunogenicity. Yet, maturation of pancreatic endoderm to endocrine cells enforces resistance to complement-mediated cytotoxicity. This implies that, while simply preventing inflammation around transplantation of pancreatic endoderm may suffice initially, the maturing graft may need protection from cellular immune attack. Therefore, protecting grafts by cell-impermeable macro-encapsulation could be a successful strategy in clinical implementation. Alternatively, or in addition, the hypoimmunogenicity of the initially engrafted pancreatic endoderm may create a window of opportunity in which to induce graft-specific tolerance.

## Electronic supplementary material

Below is the link to the electronic supplementary material.

ESM 1 (PDF 435 kb)

## Abbreviations

ADCC: Antibody-dependent cellular cytotoxicity
APC: Allophycocyanin
B-LCL: B lymphocytes
CMV: Cytomegalovirus
CTL: Cytotoxic T lymphocyte
DAF: Decay-accelerating factor
EBV: Epstein–Barr virus
EF1α: Elongation factor 1α
FITC: Fluorescein isothiocyanate
hESC: Human embryonic stem cell
hESC-EC: hESC-derived endocrine cell
hESC-PE: hESC-derived pancreatic endodermal cell
MCP: Membrane cofactor protein
MIP: Membrane attack complex-inhibitory protein
NK: Natural killer
PBMC: Peripheral blood mononuclear cell
PPI: Preproinsulin
PTEC: Primary tubular epithelial cell
Th: T helper
