# Supplementary material for: Immunogenicity of human embryonic stem cell-derived beta cells
Source: Diabetologia. 2016 Oct 27;60(1):126–33. doi: 10.1007/s00125-016-4125-y (PMC6518073; doi:10.1007/s00125-016-4125-y)
Supplement: Supplementary file 1 — (PDF 435 kb) [file 125_2016_4125_MOESM1_ESM.pdf]

ESM Fig. 1: Figure 2 statistics supplement

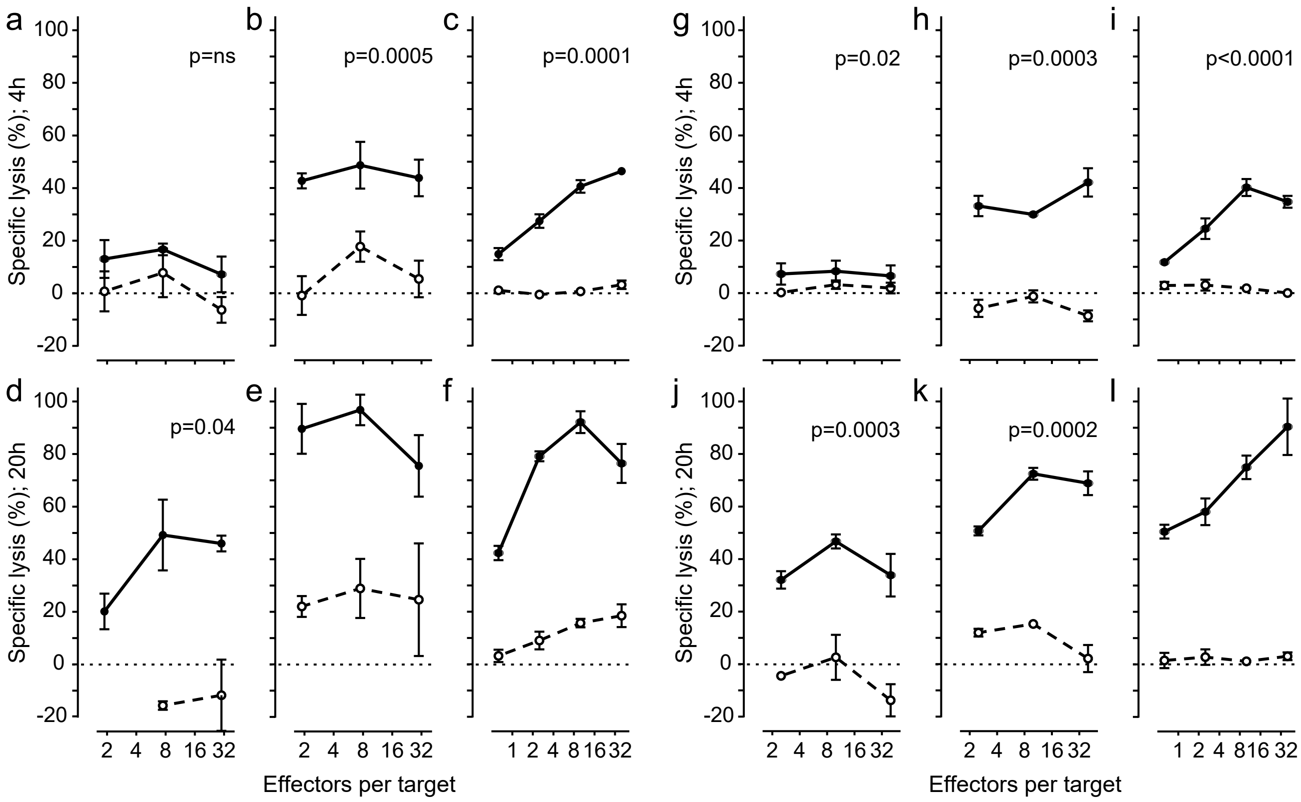

Alloreactive and virus specific CTLs can target hESC-PEs and differentiated hESC-ECs.

ESC-PEs (a, b, d, e, g, h, j, k) and hESC-ECs (c, f, i, l) expressing HLA-A1 were labelled with ^{51}Cr and incubated with alloreactive CTLs targeting HLA-A1 (black circles, solid line) or targeting third party HLA-A2 (white circles, dashed line) (a-f) and virus specific CTLs recognising CMV peptide in HLA-A1 on peptide pulsed cells (black circles, solid line) but without peptide (white circles, dashed line) (g-l). Specific lysis after 4 h (a-c, g-i) and 20 h (d-f, j-l) was calculated relative to spontaneous lysis without T cells and chemically-induced maximum lysis. Inflammation was mimicked (b, e, h, k) by pre-incubation with IFNγ (1000 IU/ml), upregulating HLA expression.

ESM Fig. 2: Figure 4 statistics supplement

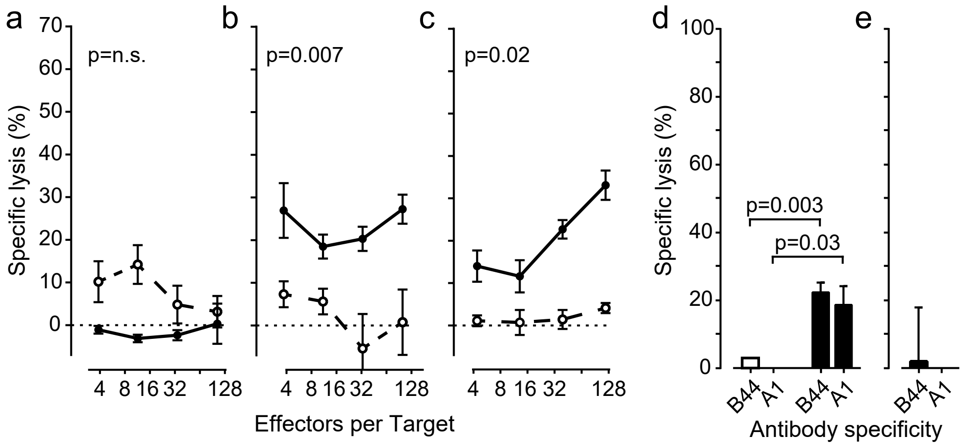

Alloreactive antibodies induce cellular cytotoxicity, but differentiated hESC-ECs resist complement dependent cytotoxicity.

(a–c) hESC-PEs (a, b) and differentiated hESC-ECs (c) were labeled with 51Cr and incubated with specific (black circles, solid line) and non-specific (white circles, dashed line) alloreactive antibodies and peripheral blood lymphocytes for 6 h. Chromium release was measured and specific lysis was calculated relative to spontaneous lysis without T cells and chemical induced maximum lysis. Inflammation was mimicked by pre-incubation with IFNγ (1000 IU/ml), upregulating HLA expression (b). (d, e) hESC-PEs (d) and hESC-ECs (e) were incubated with serum containing HLA specific antibodies to A1 and B44 for 1 h and then for 1 h with rabbit complement. Lysis was assessed by propidium iodide staining with automated microscopy measurement and specific lysis calculated relative to cell death without specific antibodies and parallel specific lysis of lymphocyte controls. Inflammation was mimicked by pre-incubation with IFNγ (1000 IU/ml), upregulating HLA expression (black bars); white bars, no pre-incubation with IFNγ. hESC-ECs were only tested after IFNγ pre-incubation.
